# Radiomics models to predict axillary lymph node metastasis in breast cancer and analysis of the biological significance of radiomic features

**DOI:** 10.3389/fonc.2025.1546229

**Published:** 2025-06-19

**Authors:** Xinhua Li, Minping Hong, Zhendong Lu, Zilin Liu, Lifu Lin, Hongfa Xu

**Affiliations:** ^1^ Oncology Center, Department of Radiology, Affiliated Hospital of Guangdong Medical University, Zhanjiang, China; ^2^ Department of Radiology, Jiaxing Hospital of Traditional Chinese Medicine Affiliated to Zhejiang Chinese Medical University, Jiaxing, Zhejiang, China

**Keywords:** axillary lymph node metastasis, radiomics, biological significance, cancer imaging archive-the cancer genome atlas, breast cancer

## Abstract

**Objectives:**

To explore the effectiveness of radiomics in predicting axillary lymph node metastasis (ALNM) and the relationship between radiomics features and genes.

**Method:**

The 379 patients with breast cancer (186 ALNM-positive and 193 ALNM-negative) recruited from three hospitals were divided into the training (n=224), testing (n=96), and validation (n=59) cohorts. The Cancer Imaging Archive-The Cancer Genome Atlas (TCIA-TCGA) group included 107 patients with breast cancer. A total of 1888 intratumoral and peritumoral radiomics features were extracted from DCE-MRI sequences. Radiomics models were established using a multivariate regression algorithm for each region and their combinations. Clinical and combined nomogram models integrating the Radscore with clinical risk factors were constructed. The biological significance of the radiomic features was analyzed by combining the TCIA database.

**Results:**

The area under the ROC curve (AUC) of radiomics model in the external validation was 0.760 (95% confidence interval [CI]: 0.626-0.874). The performance of the nomogram combined model (AUC: 0.818; 95% CI:0.702-0.916) surpassed those of both the radiomics and clinical models (AUC: 0.753; 95% CI: 0.630-0.869). Additionally, the DCA results demonstrated the usefulness of the radiomics and nomogram model.

**Conclusion:**

MRI-based radiomics has the potential to predict the ALNM status in patients with invasive breast cancer. Additionally, radiogenomic analysis demonstrated a correlation between radiomic features and the immune microenvironment.

## 1 Introduction

Breast cancer (BC) is the most prevalent cancer in women and ranks as the second primary factor behind cancer-related fatalities on a global scale (1). Axillary lymph node metastasis (ALNM) considerably impacts the staging, diagnosis, treatment, and prognosis of patients with BC. Nevertheless, the current techniques employed to diagnose ALNM, such as axillary lymph node dissection and sentinel lymph node biopsy, present potential complications (2). Furthermore, imaging modalities such as ultrasonography, mammography, and magnetic resonance imaging (MRI) fail to accurately detect atypical ALNM, thus frequently leading to unwarranted surgeries or biopsies and impairing overall treatment effectiveness (3, 4).

Hence, innovative methods to precisely and noninvasively predict ALNM should be explored. Among potential methods, radiomics is a noninvasive predictive model that can reflect biological cell- and molecular-level characteristics by analyzing quantitative features in medical images (5). Radiomics-based biomarkers have shown promising results in BC diagnosis and prognosis assessment (6). However, these biomarkers are often data-driven and lack biological explanations, considerably limiting their clinical application (7).

Therefore, it is essential to investigate the biological mechanisms underlying radiomic characteristics, specifically the relationships between radiomic traits, genes, and the tumor microenvironment. The integration of data from The Cancer Genome Atlas (TCGA) and The Cancer Imaging Archive (TCIA), which combines genetic information from TCGA with corresponding MR images from TCIA, is a valuable resource (8). This integration can unveil the intricate relationship between radiomic characteristics and genes, providing clinicians with insights into the biological significance of radiomic features and enhancing our understanding of BC at the molecular level.

Therefore, this study aimed to develop a radiomics model that predicts the ALNM status in patients with BC using data from multiple research centers. Furthermore, we combined this model with the TCIA database to examine the relationship between radiomic features, genes, and the tumor microenvironment.

## 2 Methods

### 2.1 Data sources

**Figure 1:**
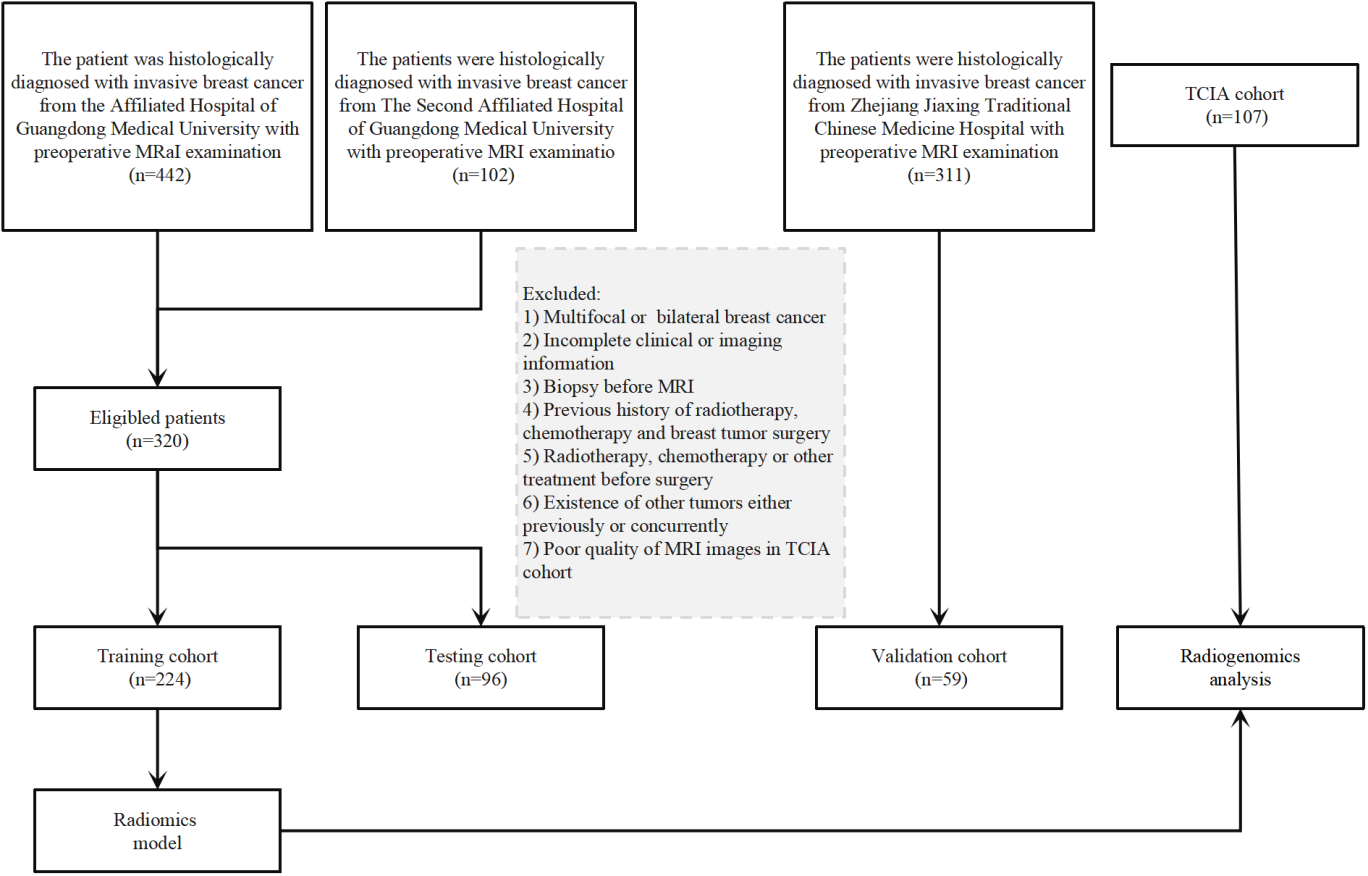
Flowchart of patient inclusion and exclusion.

A total of 486 patients with BC were included in the study from three hospitals and the TCGA-TCIA public databases. The patients were divided into two groups based on their ALNM status: ALNM-positive and ALNM-negative. The criteria for patient inclusion and exclusion are provided in the **Supplementary Material**, and the study flowchart can be found in **Figure 1**.

The following clinical information was collected from the three hospitals: age, clinical T (cT) stage, enhancement characteristics (even or uneven), maximum lesion diameter, lesion boundary, lesion morphology, spiculation, rim enhancement, and MRI assessment of ALNM status. The TCGA-TCIA database contains mRNA expression data for patients with BC. The present study received approval from three institutional review boards, which waived the requirement for obtaining written informed consent given the retrospective study design.

The multicenter study received approval from three institutional review boards, which waived the requirement for obtaining written informed consent given the retrospective study design (study proctol: SL-2022–0369; PJKT2023-087; PJKT-2024–009).

### 2.2 MRI acquisition

Dynamic contrast enhancement MRI (DCE-MRI) scans were conducted using 1.5-T or 3.0-T scanners at the three institutions. The DCE-MRI protocol involved injecting a contrast agent through the median elbow vein at a dose of 0.2 mmol/kg using a high-pressure syringe. The contrast agent was injected at a rate of 2.0 mL/s, followed by the injection of 20 mL of physiological saline at the same rate. Images were collected at six different time phases, with a mask used in the first phase. The specific sequence parameters for DCE-MRI can be found in the **Supplementary Material**.

### 2.3 Image segmentation

Considering the high clarity of the lesions in DCE-MRI phase III, we exported these images from the picture archiving and communication system in the Digital Imaging and Communications in Medicine format. To reduce the impact of different voxel spacings and reconstruction layer thicknesses on radiomic features, we resampled all images using a linear interpolation algorithm before image segmentation. Two radiologists (L.Z.L. and L.Z.D.) segmented the region of interest (ROI) along the boundaries of the tumors in each two-dimensional MRI slice using the ITK-SNAP software. Each ROI was evenly expanded by 5 mm using Python software. Further review was conducted by a third radiologist (L.X.H.), any disagreements were discussed, and a consensus was reached.

Resegmentation was performed in a random selection of 30% of the cases after 1 month, and the stability of the delineated ROIs was evaluated using the intraclass correlation coefficient (ICC). An ICC >0.75 indicated high repeatability and consistency of the ROI segmentation.

### 2.4 Radiomic feature extraction and selection

**Figure 2:**
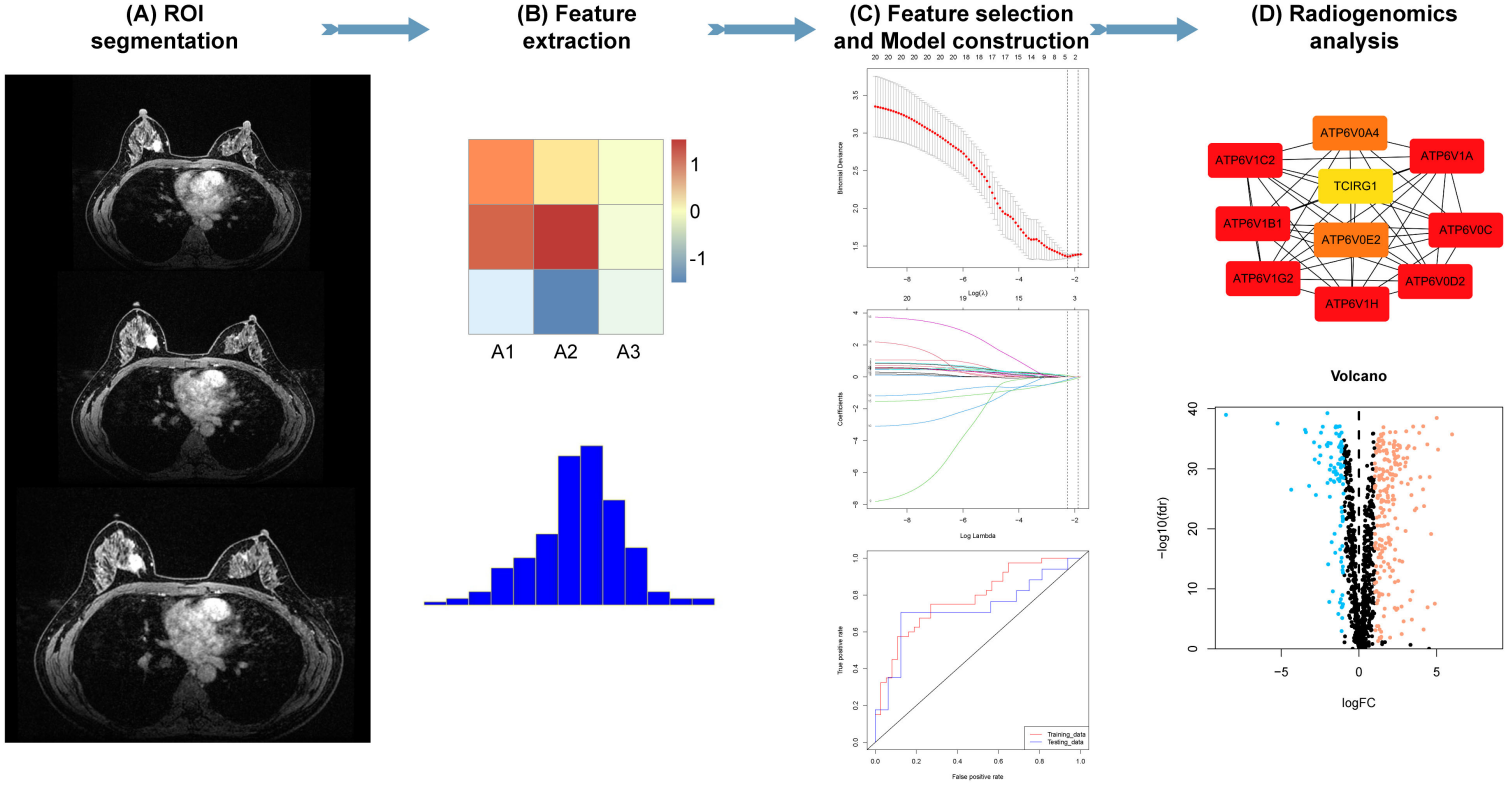
Radiomics analysis. **(A, B)** Tumor segmentation and radiomic feature extraction on magnetic resonance imaging (MRI) scans. **(C)** Feature selection and radiomics model construction. **(D)** Association of radiomic features with gene signatures.

The radiomics workflow used in this study is shown in **Figure 2**. The patients were divided into four cohorts: a training cohort (70% of patients in centers 1 and 2, n=224), internal testing cohort (30% of patients in centers 1 and 2, n=96), external validation cohort (center 3, n=59), and a TCGA-TCIA cohort (n=107). The TCGA-TCIA cohort included MR images of the patients and their corresponding transcriptomic data (https://portal.gdc.cancer.gov/).

Two groups of radiomic features, including original, square, and wavelet filtering, and a total of 1888 features were extracted using the pyradiomics module in 3D-Slicer. The radiomic features were standardized using the z-score algorithm for further analysis. To mitigate the influence of redundant features on model development, we initially employed the Mann-Whitney U test and correlation analysis to minimize the dimensionality of the attributes within and adjacent to the tumor. Subsequently, the elastic network algorithm was used to further filter radiomic attributes with predictive potential. Finally, by employing the same elastic network algorithm, we performed additional screening to obtain the combined radiomic attributes encompassing the tumor and its surroundings.

### 2.5 Construction of the models and comparison of the combined model with manual diagnosis of ALNM

Clinical model: We performed univariate and multiple logistic analyses to assess the significance of the associations between clinical factors and ALNM outcomes and constructed a clinical model based on these results.

Radiomics models: We also constructed radiomics models for evaluating ALNM in BC based on intratumoral and combined intratumoral and peritumoral radiomic features by multiple machine learning algorithms.

Nomogram model: The features of the radiomics model with the best predictive performance and linear combinations were calculated using radiomics scores (Radscores). The combined clinical risk factors with predictive values and Radscores were determined using a logistic regression algorithm to build a nomogram model. Specifically, scores were assigned to each independent variable based on the magnitude of the partial regression coefficients in the multifactor model, and the total score was obtained by summing the scores of the independent variables. This total score was then used to estimate the predictive situation of lymph node metastasis in breast cancer. The clinical advantages of clinical, radiomic, and nomogram models were assessed using decision curve analysis (DCA).

We further compared the accuracy of the combined model in diagnosing ALNM with radiologists’ assessment of ALNM on MR images (MRI_ALNM model) to improve clinicians’ understanding of the radiomics model’s assessment of ALNM.

### 2.6 Radiogenomic analysis of ALNM-related low-risk and high-risk groups

To investigate the correlation between radiomic and genomic features of BC, we downloaded RNA-seq data of 107 BC from the TCGA database in January 2024 and calculated Radscore by radiomics model. The patients in the TCIA dataset (n=107) were divided into low-risk and high-risk groups based on their median of Radscores. We used the “GSEA” package in R to detect differentially expressed genes (DEGs) in the sequencing data of these two groups of patients. To identify the signaling pathways associated with the DEGs, we conducted gene set enrichment analysis (GSEA) using the Gene Ontology (GO) database, with the corrected P-value threshold set at <0.01. We performed a hierarchical cluster analysis to investigate the functions of these pathways. To evaluate the variance in immune cell infiltration between the predicted ALNM-related low-risk and high-risk groups, we applied xCell, an analysis tool that utilizes gene expression data to estimate the abundance of individual cell types in mixed cell populations (9).

### 2.7 Statistical analysis

R version 4.1.0 was utilized to perform statistical analysis and generate plots. Various statistical tests were used in comparing the data of the two cohorts. These tests included the unpaired Student’s t-test, Mann-Whitney U test, chi-square test, and Fisher’s exact test. To evaluate the validity of the radiomics, clinical, and combined models, we calculated the area under the receiver operating characteristic (ROC) and curves (AUCs) along with their corresponding 95% confidence intervals (CIs). Unless stated otherwise, statistical significance was considered when P < 0.05.

## 3 Results

### 3.1 Patient clinical characteristics

The ages of patients in the three medical centers and the TCIA cohort ranged from 21 to 85 years. Among the patients from the three medical centers, 186 (49%) and 193 (51%) were pathologically confirmed to be ALNM-positive and ALNM-negative, respectively. This information could not be used from the TCIA cohort. In the training, internal testing, and external validation cohorts, ALNM-positive BCs had a longer diameter, greater spiculation, and higher T stage than those of ALNM-negative BCs (**Table 1**).

**Table 1:** Patient baseline.

Cohort (number, N)		External validation cohort (N=59)			Internal Testing cohort (N=96)			Training cohort (N=224)		
Parameters	Levels	ALNM-negative (N=32)	ALNM-positive (N=27)	P value	ALNM-negative (N=48)	ALNM-positive (N=48)	p value	ALNM-negative (N=113)	ALNM-positive (N=111)	P value
Lesion boundary	Clear	29 (90.6%)	24 (88.9%)	1.000	24 (50%)	27 (56.2%)	.683	62 (54.9%)	67 (60.4%)	.486
	Unclear	3 (9.4%)	3 (11.1%)		24 (50%)	21 (43.8%)		51 (45.1%)	44 (39.6%)	
Lesion morphology	Regular	12 (37.5%)	3 (11.1%)	.043	16 (33.3%)	13 (27.1%)	.657	38 (33.6%)	34 (30.6%)	.736
	Irregular	20 (62.5%)	24 (88.9%)		32 (66.7%)	35 (72.9%)		75 (66.4%)	77 (69.4%)	
Enhanced pattern	Mass	30 (93.8%)	15 (55.6%)	.002	36 (75%)	35 (72.9%)	1.000	79 (69.9%)	88 (79.3%)	.145
	Non_mass	2 (6.2%)	12 (44.4%)		12 (25%)	13 (27.1%)		34 (30.1%)	23 (20.7%)	
Rim enhancement	Without	22 (68.8%)	2 (7.4%)	<.001	39 (81.2%)	32 (66.7%)	.163	88 (77.9%)	80 (72.1%)	.396
	With	10 (31.2%)	25 (92.6%)		9 (18.8%)	16 (33.3%)		25 (22.1%)	31 (27.9%)	
Enhancement characteristic	Without	23 (71.9%)	21 (77.8%)	.827	30 (62.5%)	19 (39.6%)	.041	56 (49.6%)	48 (43.2%)	.416
	With	9 (28.1%)	6 (22.2%)		18 (37.5%)	29 (60.4%)		57 (50.4%)	63 (56.8%)	
MR_ALNM	Negative	22 (68.8%)	10 (37%)	.030	41 (85.4%)	20 (41.7%)	<.001	95 (84.1%)	54 (48.6%)	<.001
	Positive	10 (31.2%)	17 (63%)		7 (14.6%)	28 (58.3%)		18 (15.9%)	57 (51.4%)	
cT	T1-2	23 (71.9%)	14 (51.9%)	.189	44 (91.7%)	34 (70.8%)	.019	104 (92%)	89 (80.2%)	.018
	T3-4	9 (28.1%)	13 (48.1%)		4 (8.3%)	14 (29.2%)		9 (8%)	22 (19.8%)	
Spiculation	Without	26 (81.2%)	7 (25.9%)	<.001	30 (62.5%)	18 (37.5%)	.025	74 (65.5%)	53 (47.7%)	.011
	With	6 (18.8%)	20 (74.1%)		18 (37.5%)	30 (62.5%)		39 (34.5%)	58 (52.3%)	
Age	Mean ± SD	54.16 ± 10.72	51.63 ± 8.60	.329	49.12 ± 10.38	50.31 ± 12.32	.611	49.00 (41.00 to 59.00)	49.00 (43.00 to 55.00)	.785
Maximum lesion diameter	Median (IQR)	2.35 (1.85 to 3.90)	3.40 (2.60 to 5.35)	.007	2.30 (1.75 to 3.00)	2.80 (1.90 to 3.90)	.013	1.80 (1.40 to 2.60)	2.50 (1.80 to 3.45)	<.001

### 3.2 Performance of the ALNM prediction models

**Figure 3:**
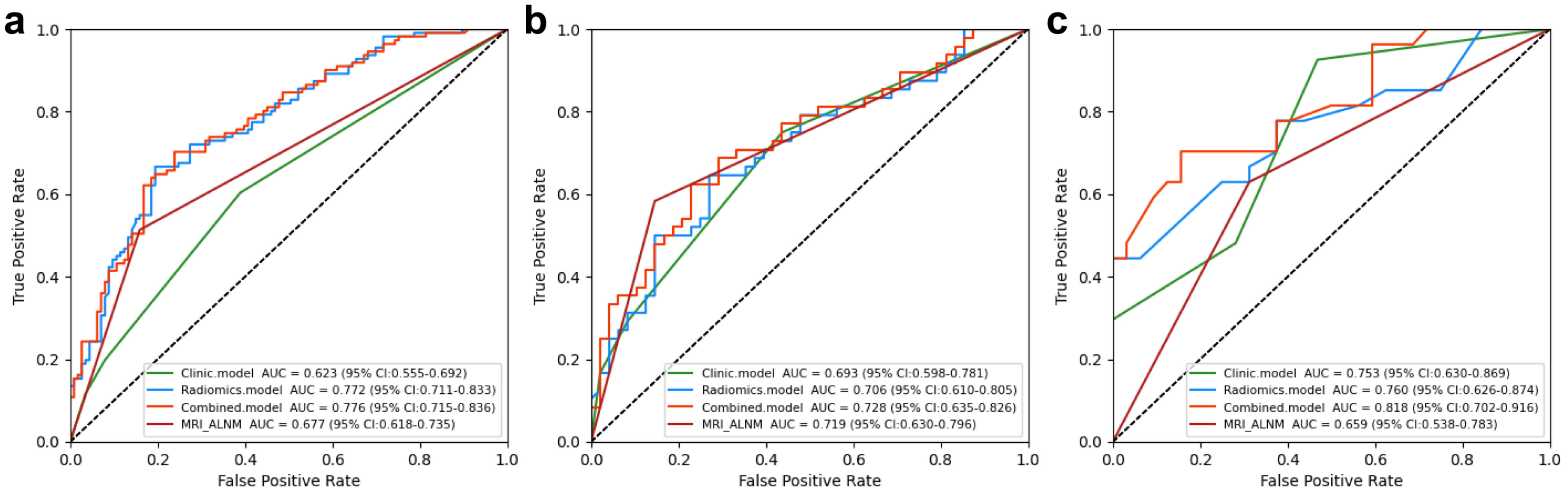
Performance of the clinical, radiomics, and nomogram models in the training **(a)**, internal testing **(b)**, and external validation **(c)** cohorts.

Clinical model: The results showed that spiculation and cT stage were separate factors that independently increased the risk of ALNM (**Table 2**). A model using these factors was developed and validated in a separate cohort. The assessment of the model’s predictive ability yielded an AUC of 0.753 (95% CI: 0.650-0.865), sensitivity of 0.926, specificity of 0.531, and accuracy of 0.712 in the external validation cohort (**Figure 3**, **Table 3**).

**Table 2:** Univariate and multivariate logistics analysis.

Dependent: label		OR (univariable)	OR (multivariable)
Age	Mean ± SD	0.99 (0.97-1.02, p=.617)	
Lesion boundary	clear		
	unclear	0.77 (0.45-1.31, p=.335)	
Lesion morphology	regular		
	unregular	1.44 (0.83-2.49, p=.196)	
Enhancement pattern	mass		
	non_mass	0.61 (0.34-1.12, p=.114)	
Spiculation	Without		
	With	2.49 (1.45-4.26, p<.001)	2.53 (1.46-4.39, p<.001)
Rim enhancement	Without		
	With	1.57 (0.85-2.88, p=.146)	
Enhancement characteristics	Without		
	With	1.61 (0.95-2.73, p=.080)	
cT stage	T1-2		
	T3-4	2.99 (1.36-6.58, p=.006)	3.09 (1.38-6.92, p=.006)

SD, Standard Deviation.

**Table 3:** Performance of clinical, radiomics, nomgram and MR_ALNM model.

Cohort	Model	AUC (95% CI)	Accuracy	Sensitivity	Specificity
Training cohort	Clinic.model	0.623 (0.555-0.692)	0.607	0.604	0.611
	Radiomics.model	0.772 (0.711-0.833)	0.737	0.667	0.805
	Combined.model	0.776 (0.715-0.836)	0.732	0.703	0.761
	MRI_ALNM	0.677 (0.618-0.735)	0.679	0.514	0.841
Internal testing cohort	Clinic.model	0.693 (0.598-0.781)	0.656	0.75	0.562
	Radiomics.model	0.706 (0.610-0.805)	0.646	0.646	0.646
	Combined.model	0.728 (0.635-0.826)	0.656	0.729	0.583
	MRI_ALNM	0.719 (0.630-0.796)	0.719	0.583	0.854
External validation cohort	Clinic.model	0.753 (0.630-0.869)	0.712	0.926	0.531
	Radiomics.model	0.760 (0.626-0.874)	0.678	0.667	0.688
	Combined.model	0.818 (0.702-0.916)	0.661	0.704	0.625
	MRI_ALNM	0.659 (0.538-0.783)	0.661	0.63	0.688

**Figure 4:**
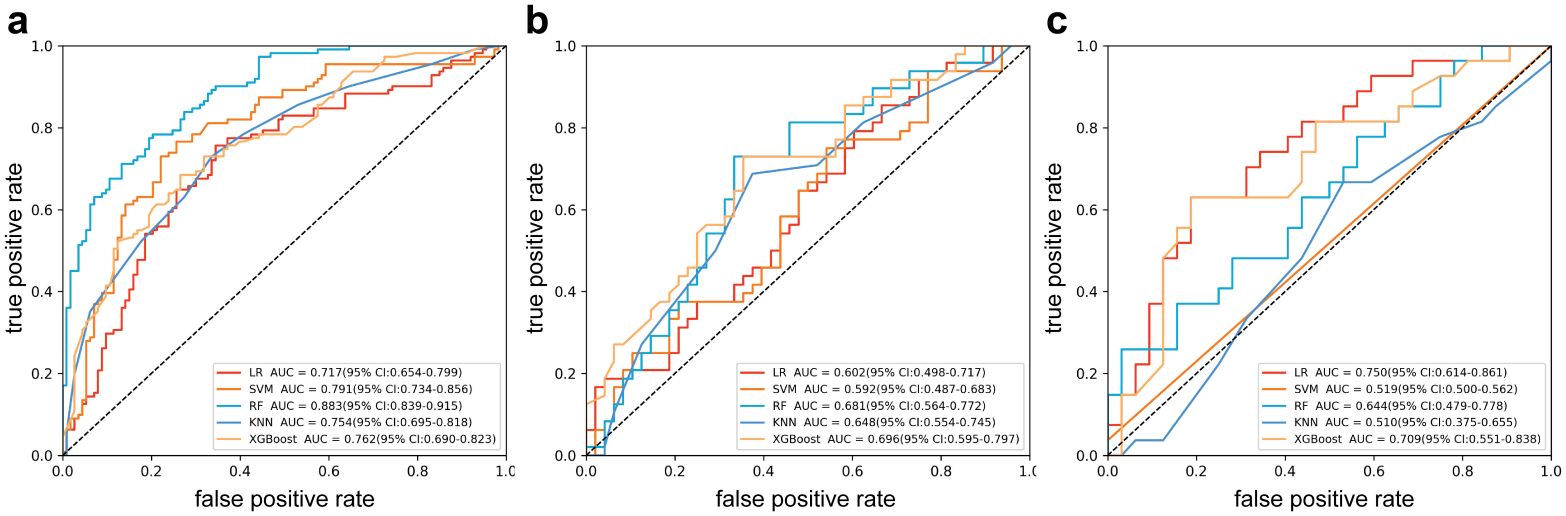
Receiver operating characteristic curves of intratumor radiomics models (multiple machine learning algorithms). **(a)** Training cohort; **(b)** internal testing cohort; **(c)** external validation cohort.

**Figure 5:**
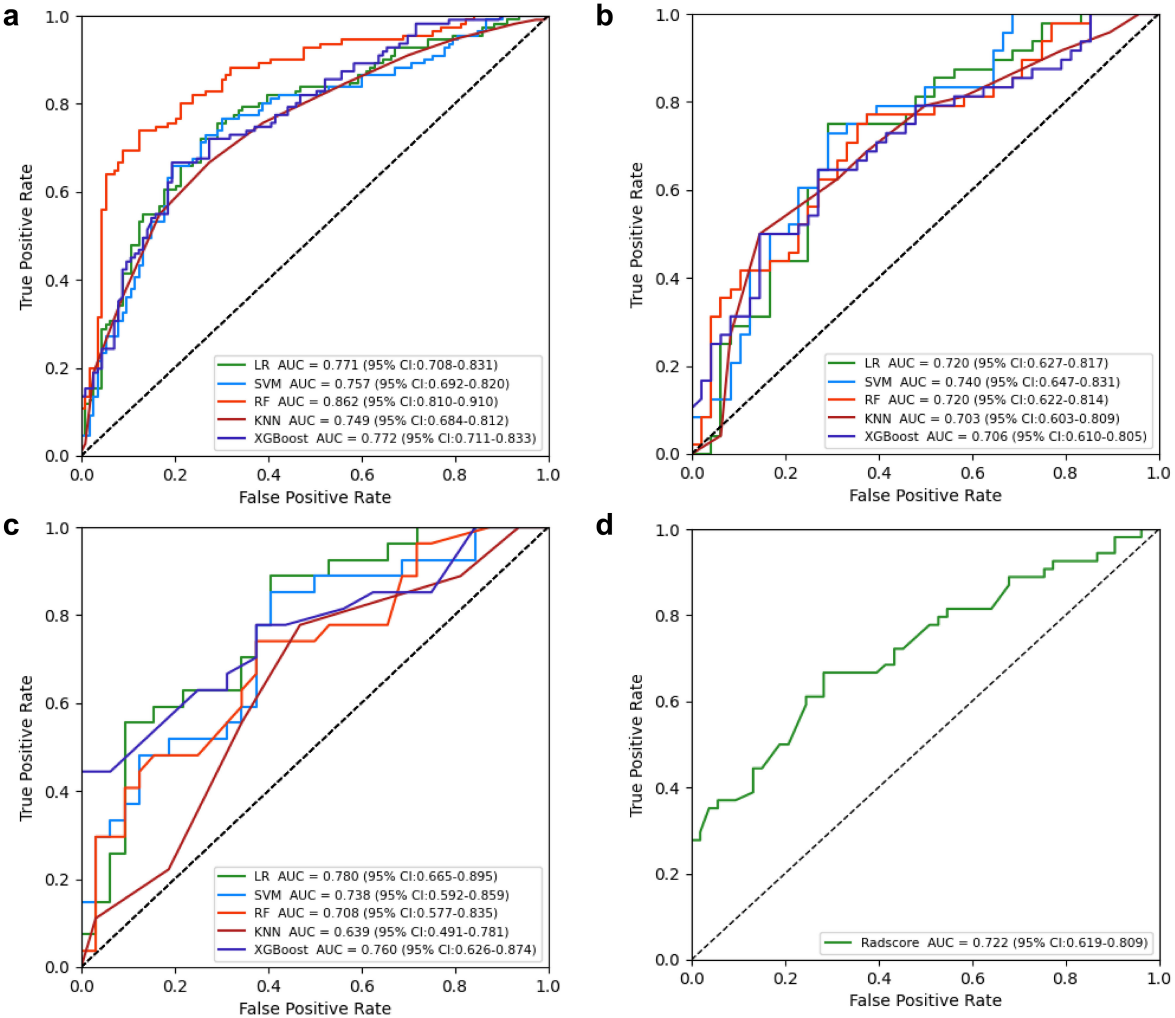
Receiver operating characteristic (ROC) curves of intratumor radiomics models (multiple machine learning algorithms). **(a)** Training cohort; **(b)** internal testing cohort; **(c)** external validation cohort. **(d)** ROC curves of Radscore model in TCIA cohort.

Radiomics model: The **Supplementary Material** provides a detailed overview of the radiomic features utilized in three radiomics models: intratumoral and a combination of intratumoral with peritumoral 5 mm. Among these models, the intratumoral model exhibited an AUC value of 0.709 (95% CI: 0.551-0.838), indicating a sensitivity of 0.741 and a specificity of 0.562 in the external validation cohort (**Table 4**, **Figure 4**). The most accurate predictive performance was achieved by the model that merged intratumoral and peritumoral 5 mm features (XGBoost algorithm) integrating all aspects of the performance of the model. The external validation cohort displayed an AUC of 0.760 (95% CI: 0.626-0.874), accompanied by a sensitivity of 0.667 and a specificity of 0.688 (**Figure 5**, **Table 5**). Furthermore, the TCIA cohort displayed an AUC of 0.722 (95% CI: 0.619-0.809), accompanied by a sensitivity of 0.630 and a specificity of 0.717 (**Figure 5d**).

**Table 4:** Performance of intratumor radiomics model.

Group	Model	AUC (95% CI)	Accuracy	Sensitivity	Specificity
Training cohort	LR	0.717 (0.654-0.799)	0.705	0.757	0.655
	SVM	0.791 (0.734-0.856)	0.754	0.766	0.743
	RF	0.883 (0.839-0.915)	0.79	0.784	0.796
	KNN	0.754 (0.695-0.818)	0.696	0.73	0.664
	XGBoost	0.762 (0.690-0.823)	0.71	0.685	0.735
Internal Testing cohort	LR	0.602 (0.498-0.717)	0.583	0.75	0.417
	SVM	0.592 (0.487-0.683)	0.583	0.708	0.458
	RF	0.681 (0.564-0.772)	0.646	0.75	0.542
	KNN	0.648 (0.554-0.745)	0.594	0.708	0.479
	XGBoost	0.696 (0.595-0.797)	0.677	0.729	0.625
External validation cohort	LR	0.750 (0.614-0.861)	0.61	0.222	0.938
	SVM	0.519 (0.500-0.562)	0.458	0.571	0.433
	RF	0.644 (0.479-0.778)	0.576	0.593	0.562
	KNN	0.510 (0.375-0.655)	0.525	0.481	0.562
	XGBoost	0.709 (0.551-0.838)	0.644	0.741	0.562

LR, Logistic Regression; KNN, K-Nearest Neighbors; XGBoost, EXtreme Gradient Boosting.

**Table 5:** Performance of intratumoral combined peritumoral radiomics models.

Cohort	Model	AUC (95% CI)	Accuracy	Sensitivity	Specificity
Training cohort	LR	0.771 (0.708-0.831)	0.732	0.757	0.708
	SVM	0.757 (0.692-0.820)	0.732	0.766	0.699
	RF	0.862 (0.810-0.910)	0.808	0.739	0.876
	KNN	0.749 (0.684-0.812)	0.696	0.667	0.726
	XGBoost	0.772 (0.711-0.833)	0.737	0.667	0.805
Internal Testing cohort	LR	0.720 (0.627-0.817)	0.656	0.812	0.5
	SVM	0.740 (0.647-0.831)	0.677	0.792	0.562
	RF	0.720 (0.622-0.814)	0.688	0.75	0.625
	KNN	0.703 (0.603-0.809)	0.656	0.688	0.625
	XGBoost	0.706 (0.610-0.805)	0.667	0.646	0.688
External validation cohort	LR	0.780 (0.665-0.895)	0.61	0.222	0.938
	SVM	0.738 (0.592-0.859)	0.661	0.704	0.625
	RF	0.708 (0.577-0.835)	0.661	0.481	0.812
	KNN	0.639 (0.491-0.781)	0.644	0.778	0.531
	XGBoost	0.760 (0.626-0.874)	0.678	0.667	0.688
TCIA cohort	XGBoost	0.722 (0.619-0.809)	0.673	0.63	0.717

LR, Logistic Regression; KNN, K-Nearest Neighbors; XGBoost, EXtreme Gradient Boosting.

**Figure 6:**
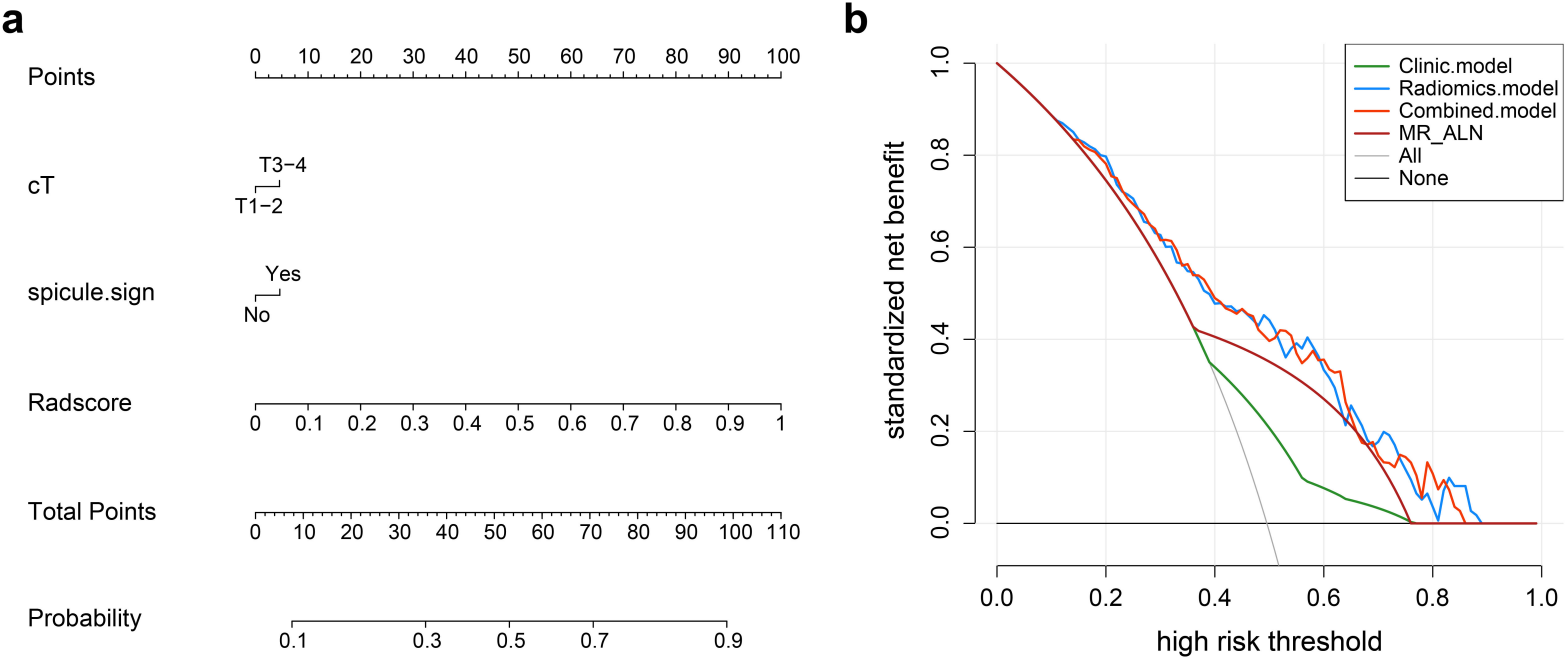
**(a)** Nomogram for predicting axillary lymph node metastasis (ALNM). The nomogram combines the radiomics score (Radscore) with cT stage and spiculation in the training cohort. The probability value for each patient with breast cancer with ALNM is marked on each axis. **(b)** Decision curve analysis of the clinical, radiomics, and nomogram models.

Nomogram model: Using multivariate regression analysis, a nomogram model was created by integrating two risk factors associated with ALNM, namely, spiculation and cT stage, along with the Radscore. The nomogram model showed an AUC of 0.818 (95% CI: 0.706-0.904), sensitivity of 0.741, specificity of 0.625, and accuracy of 0.678 in the external validation cohort (**Figure 6**, **Table 3**). The results of DCA shown in **Figure 5b** suggest that the combined model may yield greater clinical advantages compared to the radiomic and clinical models for identifying ALNM.

### 3.3 Performance of the radiomics model compared with that of radiologists

In the validation cohort, the MRI-ALNM model had an AUC of 0.659 (95% CI: 0.538-0.783), sensitivity of 0.630, and specificity of 0.688 (**Figure 3**, **Table 3**), which were lower than those of the combined model. This result further demonstrated that radiomics models can help improve the accuracy of ALNM assessments in clinical practice.

### 3.4 Biological significance of radiomic characteristics

**Figure 7:**
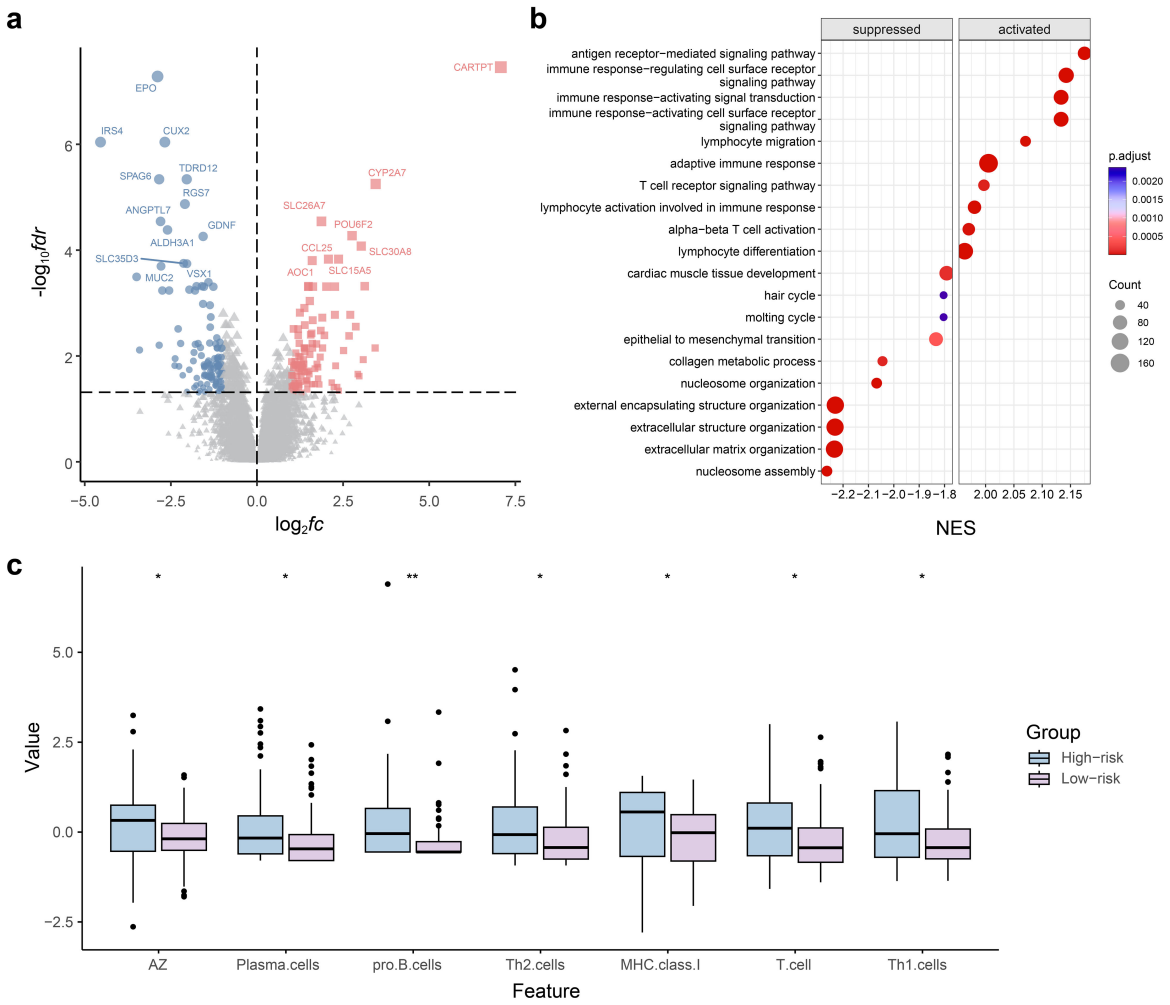
Radiogenomics analysis for predicting axillary lymph node metastasis (ALNM) in the radiomics model. **(a)** Volcano plots of gene expression profile data in The Cancer Imaging Archive (TCIA): analysis of differentially expressed genes (DEGs) between ALNM-positive and ALNM-negative groups. **(b)** Gene set enrichment analysis of predicted DEGs based on the Gene Ontology database. **(c)** Violin plot of the results of the immunoinfiltration analysis.

The DEG analysis findings demonstrated a total of 225 DEGs, with 116 upregulated and 109 downregulated genes identified in the radiomics model for ALNM. This was observed in the comparison of the predicted low- and high-risk groups (**Figure 7a**). Upon conducting GO analysis, we discovered that the upregulated genes in the ALNM-positive population, as predicted by the radiomics model, exhibited significant enrichment in immune and inflammatory signaling pathways. These pathways encompassed immune cell activation and regulation of leukocyte chemical tendencies (**Figure 7b**). Furthermore, the expression levels of B, T, and natural killer cells were notably high in the anticipated high-risk group for ALNM. This observation sheds light on the strong correlation between the immune microenvironment of patients with BC and ALNM. Consequently, the immune scores for both groups were calculated, and immune infiltration was comprehensively analyzed. The analysis results divulged substantial disparities in various components of the immune microenvironment, including plasma cells, Th2 cells, and Th1 cells, when comparing the high- and low-risk ALNM groups (**Figure 7c**).

## 4 Discussion

We developed a nomogram model as an innovative method for the noninvasive prediction of ALNM. This model utilizes intratumoral and peritumoral DCE-MRI radiomic features in conjunction with clinical and radiological features to predict the lymph node status. To evaluate the objective performance of the combined model, it was compared with the diagnostic efficacy of a highly experienced radiologist for determining ALNM on breast MR images. The findings revealed that the combined model outperformed manual diagnostic ability in predicting nodal status, with the highest level of predictive performance. Furthermore, the relationship between radiomic characteristics, genes, and tumor microenvironment was investigated using the TCGA-TCIA database. This investigation aimed to enhance the biological interpretability of the radiomics model.

Univariate and multiple logistic analyses showed that cT stage and spiculation were independent risk factors for ALNM, which is consistent with the results of previous studies. cT stage and spiculation indicate that BC is more aggressive and prone to ALNM (10–12). However, the performance of the clinical model constructed based on these two risk factors was relatively low, indicating the limited practical application value of this clinical model.

Our results demonstrated that the DCE-MRI-based radiomics prediction model performed well. Using the radiomic features extracted from DCE-MR images, we can perform a more accurate analysis of the diagnosis and prognosis of BC in multiple dimensions, including tumor hemodynamic characteristics, tissue heterogeneity, and internal structure complexity (13–15). In addition, our model is based on multicenter MRI data and showed a higher performance than manual diagnosis, indicating that our model has good generalization ability and clinical practicability.

Radiomic features around tumors have attracted increasing attention in recent years. Sun et al. reported that ultrasound radiomic features, including peritumoral regions, could improve the performance of predictive models for ALNM in BC (16). Chen et al. constructed a deep-learning model to predict sentinel lymph node metastasis; their analysis of the prediction process revealed that the model focused on areas within and around the tumor (17). These studies highlight the significance of peritumoral radiomic features that encapsulate a wealth of information on the microenvironment and heterogeneity of BC (18). This information is critical and should not be overlooked. Our results showed that radiomics models combining intratumoral and peritumoral 5 mm features had better predictive performance than models constructed based on intratumoral features, further confirming this observation. The Radscore of our radiomics model successfully stratified the risk of ALNM-positive and ALNM-negative patients, indicating that it is an effective biomarker. DCA of the nomogram with a further combination of Radscore and clinical factors showed superior performance, indicating that the nomogram is highly consistent with our data and has significant clinical utility.

However, the investigations mentioned above concentrated solely on the prognostic capability of radiomics models and did not assess the biological mechanisms behind the research findings (7). This disconnect between radiomic features and biological significance inevitably limits the range of clinical applications. Recent studies have demonstrated that radiomic feature extraction can be combined with genetic analysis to create a more comprehensive model for cancer characterization (18–20). This multimodal approach helps clinicians understand radiomic features, especially in conjunction with the resources provided by the TCIA. In our study, radiogenomic analysis combined with TCIA data showed a high correlation between the Radscore and genes involved in related immune/inflammatory pathways.

Previous studies have described various immune cell distributions, blood vessels, and extracellular matrices in the intratumoral and peritumoral microenvironment of BC (20). Changes in the immune microenvironment and the formation of peritumoral microvessels may lead to tumor development and metastasis (21, 22). Therefore, several immune and inflammatory pathways related to ALNM have been used to predict BC lymph node metastasis and therapeutic effects (23). Malignancies can evade the immune system by various mechanisms, including transforming macrophages induced by tumors, inactivating T cells, generating pathogenic antibodies from B cells, and activating regulatory T cells to accelerate the colonization, expansion, and spread of tumors in the lymph nodes (24, 25). These findings confirm the bidirectional role of immune cells in combating tumors and promoting lymph node metastasis in cancer cells. According to Gu et al., BC has the potential to trigger a remarkable elevation in the ratio and count of B cells prior to the occurrence of lymph node metastasis. This, in turn, stimulates B cells to produce disease-causing antibodies that specifically target the tumor antigen HSPA4. Consequently, this mechanism accelerates the process of lymph node metastasis in BC (26). The present study showing the upregulation of B cells and some regulatory T cells in the lymph node metastasis group is consistent with previous reports. Xia et al. demonstrated that radiomic characteristics not only mirror the genomic aspects of tumors but also offer extensive information on the tumor microenvironment, including variations in the immune microenvironment and alterations in glucose metabolism (18, 27, 28). In our study, the integration of radiogenomic analysis with TCIA data displayed a strong correlation between the radiomics score and genes engaged in immune/inflammatory pathways that are interconnected. Further analysis of immune infiltration demonstrated noteworthy variations in immune indicators present in the plasma, such as plasma cells, Th2 cells, and Th1 cells, amidst the ALNM high-risk and low-risk divisions. These findings highlight the potential biological significance of radiomic features within the tumor immune microenvironment. By extrapolating from these outcomes, we hold the belief that radiomic features have the potential to elucidate the underlying mechanism of ALNM in BC. This, in turn, can furnish a fresh theoretical foundation for the individualized treatment of BC.

## 5 Limitations

This study’s limitations can be listed as follows: First, the retrospective design inevitably introduces selection bias. Thus, prospective cohort studies are needed for more comprehensive investigations of the findings of the present study. Additionally, the sample size of patients enrolled in this study was relatively small, indicating the need for future research with larger sample sizes. Moreover, more diverse patient population should be included in the study to enhance the model’s applicability across different demographics. Prospective studies are needed to validate the models’ predictive power in real-world clinical settings. Finally, relevant experimental validation data were lacking to support the connection between radiomic features and the TCTA transcriptome; thus, further research and comprehensive validation are needed.

## 6 Conclusions

In this study, we constructed a radiomics model based on peritumoral and intratumoral features to predict BC ALNM, which showed a strong generalization ability. Moreover, we explored the biological significance of the radiomic characteristics of patients in the TCIA cohort, providing more theoretical support for the further clinical development of radiomics.

## Data Availability

The original contributions presented in the study are included in the article/**Supplementary Material**. Further inquiries can be directed to the corresponding author.
