# Supplementary material for: Radiomics models to predict axillary lymph node metastasis in breast cancer and analysis of the biological significance of radiomic features
Source: Front Oncol. 2025 Jun 19;15:1546229. doi: 10.3389/fonc.2025.1546229 (PMC12223324; doi:10.3389/fonc.2025.1546229)
Supplement: Supplementary file 1 [file DataSheet1.docx]

**Supplementary Materials**

**Section 1 Patient inclusion and exclusion criteria**

**Inclusion Criteria:**

1. Female patients aged at least 18 years with histologically confirmed invasive breast cancer.
2. Patients who had been treated with surgery and sentinel lymph node biopsy or lymph node dissection, and had pathologically confirmed ALN status.
3. Preoperative MRI scan of breast tumor was available.

**Exclusion Criteria:**

1. Multifocal or bilateral breast cancer.
2. Incomplete clinical or imaging information.
3. Biopsy or surgery about ALN before MRI.
4. Previous history of radiotherapy, chemotherapy and breast tumor surgery
5. Radiotherapy, chemotherapy or other treatment before surgery.
6. Existence of other tumors either previously or concurrently.
7. Poor quality of MRI images in TCIA cohort

**Section 2 Three hospitals and TCIA scanner and parameters**

| Scanner | Scanner I (3.0T) | Scanner II (3.0T) | Scanner III (1.5 T) | TCIA |
| --- | --- | --- | --- | --- |
| Sequence | Volume Imagine for Breast Assessment  (Vibrant) | Volume Imagine for Breast Assessment  (Vibrant) | Volume Imagine for Breast Assessment  (Vibrant) | 3D fast low-angle shot  (FLASH) |
| Orientation | Axial | Axial | Axial | Axial |
| Fat suppression | Vibrant Flex | Vibrant Flex | Vibrant Flex | NA |
| Repetition time (msec) | 6.1 | 4.3 | 4.8 | 1.38 |
| Echo time (msec) | 3.0 | 2.1 | 2.2 | 3.87-4.54 |
| Flip angle (°) | 10 | 12 | 10 | 10 |
| Field of view (mm^2^) | 330×330 | 320×340 | 370×370 | NA |
| Matrix | 448×350 | 320×320 | 256×160 | 448×(269-336) |
| Number of slices | 112 | 120 | 80 | NA |
| Slice thickness (mm) | 1.40 | 1.00 | 2.40 | 0.9-2 |
| Slice gap (mm) | 0 | 0.5 | 2.4 | NA |
| Number of averages | 1 | 1 | 2 | NA |
| Voxel size (mm) | 1.0×0.8×1.2 | 1.1×0.8×1.0 | 1.4×2.3×2.4 | NA |
| Pixel bandwidth（Hz） | 1086 | 1262 | 41.67 | NA |
| Acquisition time (min: sec) | 8:00 | 3:55 | 1:52 | NA |

**Section 3 Features Subsets**

**Table 1 intratumor radiomics features and their weights screened by elastic-logistic**

| **Features** | **Coefficient** |
| --- | --- |
| wavelet.HHL_glcm_MCC | -0.237957345 |
| wavelet.LHH_firstorder_Median | -0.126368391 |
| wavelet.LLH_glrlm_ShortRunLowGrayLevelEmphasis | -0.107318677 |
| wavelet.LHH_glcm_Correlation | -0.106199652 |
| wavelet.HLL_firstorder_Skewness | -0.048245238 |
| wavelet.HHL_gldm_SmallDependenceLowGrayLevelEmphasis | -0.032019375 |
| original_shape_SurfaceVolumeRatio | -0.030473708 |
| original_glrlm_LongRunLowGrayLevelEmphasis | -0.000965715 |
| wavelet.HHH_glszm_LargeAreaEmphasis | 0.001919373 |
| square_firstorder_Energy | 0.007824603 |
| wavelet.HHL_glszm_LargeAreaLowGrayLevelEmphasis | 0.016855539 |
| wavelet.LHH_gldm_LargeDependenceHighGrayLevelEmphasis | 0.049394474 |

**Table 2 intratumoral fusion with a peritumoral 5mm radiomics features and their weights screened by elastic-logistic**

| **Features** | **Coefficient** |
| --- | --- |
| Perit_5_wavelet.HHH_gldm_SmallDependenceLowGrayLevelEmphasis | -0.03961 |
| Tumor_original_gldm_SmallDependenceLowGrayLevelEmphasis | -0.0175 |
| Perit_5_original_shape_Sphericity | -0.01269 |
| Perit_5_wavelet.LHL_glcm_Imc2 | -0.00647 |
| Tumor_wavelet.HLH_glcm_Idmn | 0.002705 |
| Perit_5_original_shape_LeastAxisLength | 0.003726 |
| Perit_5_wavelet.HLL_glcm_Idmn | 0.011095 |
| Tumor_wavelet.LLL_glcm_JointEntropy | 0.020711 |
| Tumor_wavelet.LHL_firstorder_Median | 0.024129 |
| Tumor_wavelet.LLH_glcm_Idmn | 0.031054 |
| Perit_5_wavelet.LHL_glcm_Idmn | 0.031714 |
| Tumor_wavelet.HLL_glcm_Idmn | 0.032346 |
| Perit_5_square_glcm_Idn | 0.036014 |
| Tumor_wavelet.LHH_glcm_Idmn | 0.037576 |
| Perit_5_wavelet.HHL_glcm_Idmn | 0.043531 |
| Perit_5_original_shape_MinorAxisLength | 0.044885 |
| Tumor_wavelet.LHL_glcm_Idmn | 0.048331 |
| Tumor_original_glcm_Idn | 0.055274 |
| Perit_5_wavelet.LLH_glcm_Idmn | 0.071448 |
